# Effect of implant macro-design on primary stability: A prospective clinical study

**DOI:** 10.4317/medoral.21024

**Published:** 2016-01-31

**Authors:** Naroa Lozano-Carrascal, Oscar Salomó-Coll, Marta Gilabert-Cerdà, Nuria Farré-Pagés, Jordi Gargallo-Albiol, Federico Hernández-Alfaro

**Affiliations:** 1 PhD Student; Assistant professor of General Dentistry. Department of General Dentistry, International University of Catalonia, Barcelona, Spain; 2 PhD Student; Assistant professor on International Master of Oral Surgery. Department of Oral and Maxillofacial Surgery, International University of Catalonia, Barcelona, Spain; 3 Student of the International Master of Oral Surgery. Department of Oral and Maxillofacial Surgery, International University of Catalonia, Barcelona, Spain; 4 PhD Student; Assistant professor. Department of Oral and Maxillofacial Surgery, Department of Oral and Maxillofacial Surgery, International University of Catalonia, Barcelona, Spain; 5 PhD in Dentistry; Associate Professor and Director of the International Master in Oral Surgery. Department of Oral and Maxillofacial surgery, International University of Catalonia, Barcelona, Spain; 6 PhD in Dentistry; Professor and Chairman of the Department of Oral and Maxillofacial surgery. International University of Catalonia, Barcelona, Spain

## Abstract

**Background:**

Implant restorations have become a high predictable treatment option. Several caracteristics such as surgical technique and implant design can influence the treatment outcomes. The aim of the present study was to evaluate the influence of implant macro-design on primary stability measured with resonance frequency analysis (RFA) and insertion torque (IT).

**Material and Methods:**

A total of 47 implants divided in two groups: Test group (TI): 22 Tapered MIS® Seven implants; Control group (CI): 25 cylindrical Astra® Osseospeed implants. All implants were inserted following the manufacturers’ standard protocols. Implant primary stability was measured at the moment of implant placement by registering insertion torque values (ITv) and ISQ values by means of Osstell™ Mentor (ISQv) (Integration Diagnostic Ltd., Goteborg, Sweden).

**Results:**

In the mandible, mean ISQv for tapered implants (TI) was 71.67±5.16 and for cylindrical implants (CI) 57.15±4.83 (*p*=0.01). Mean insertion torque was 46.67±6.85 Ncm for TI and 35.77±6.72 Ncm for CI (*p*=0.01). In the maxilla, mean ISQ was 67.2±4.42 for tapered implants and 49.17±15.30 for cylindrical implants (*p*=0.01). Mean insertion torque for TI was 41.5±6.26 Ncm and for CI 39.17±6.34 Ncm (*p*>0.05). For tapered implants, no correlation could be found between implant diameter and primary stability. But for cylindrical implants there was a statistically significant correlation between implant diameter and primary stability: ITv (*p*=0.03); ISQv (*p*=0.04).

**Conclusions:**

Within the limits of the present study, tapered shaped implants achieve higher primary stability measured through ISQ and insertion torque values. Moreover, for cylindrical implants positive correlation has been established between implant diameter and primary stability.

**Key words:**Primary stability, tapered, cylindrical, conical, implant macro-design.

## Introduction

Implant restoration of partially or fully edentulous patients has become a highly reliable and predictable treatment option with high survival and success rates (1). The outcomes of different implant procedures depend on several variables including patient characteristics, surgical technique, and implant design. Another important factor is implant stability (2,3). Several non-invasive clinical test methods for determining implant stability have been described, such as visual evaluation, Ping test (percussion test which consists in tapping on implant-abutment interface with a metallic instrument), insertion torque, Periotest and resonance frequency analysis (RFA) (2,4). Despite the availability of a wide variety of stability quantifiers, only some of these have proved valid. Nowadays, insertion torque and RFA are seen as the gold standard for evaluating in vivo implant primary stability. Insertion torque quantifiers allow only a single measurement of primary stability, while RFA offers the possibility of checking in vivo implant stability at different times.

Insertion torque value, also known as cutting resistance measurement to assess bone density during implant surgery, was first described by Johansson and Strid in 1994 (2). Insertion torque is a mechanical parameter influenced by surgical procedure, implant design and bone quality. A high insertion torque means that the implant is firmly embedded in the bone and mechanically stable. Some authors have found that insertion torque values above 32 Ncm are an indication of adequate primary stability (5). RFA consists of applying a bending load that mimics clinical loading and direction; it provides information about the stiffness of the implant-bone junction. The value produced is a combination of bone implant contact and bone density around the implants. RFA instruments perform a quick and simple measurement, the result of measurement is presented within a parameter called implant stability quotient (ISQ). The ISQ ranges from 1 (low stability) to 100 (high stability) (6-8).

Implant stability is defined as the absence of movement at the moment of measurement. This factor can be measured at the moment of implant placement (primary stability) or once the osseointegration process is underway (secondary stability). Both parameters are interrelated positively. Traditionally, a high primary stability was associated with expectation of good secondary stability, which would ensure the likelihood of implant success and osseointegration. Consequently, poor primary stability was thought to be one of the major causes of implant failure. Primary implant stability is influenced by many factors including local bone quality and quantity, and implant macro-design (3,9,10). In this context, there is some controversy about which implant design achieves better implant stability. Implant stabilization is an important parameter in reducing fibrous tissue formation around implants; according to the literature, maximum acceptable micromovement is between 50 and 150μm. Some authors report that early failure may be caused by poor implant stability, so implant primary and secondary stability are considered key factors for implant success (5).

The aim of the present study was to evaluate clinical primary stability, by means of resonance frequency analysis and insertion torque, to discover whether implant macro-design has any influence on primary stability. It also set out to determine any relationship between implant diameter and primary stability.

## Material and Methods

The present study was conducted at the School of Dentistry og the Universitat Internacional de Catalunya, between September 2008 and April 2009. The University Ethical Committee for Clinical Research approved the study protocol (B-44-EFP-08). All patients gave their informed consent in writing to take part in the study.

- Inclusion criteria:

● Patients aged over 18 years, of either gender, and presenting adequate medical conditions to undergo implant surgery.

● Fully or partially edentulous patients attending the School of Dentistry, due for rehabilitation with dental implants, with no dental extractions performed at least 3 months before surgery.

● CT scans that showed sufficient bone volumes to allow implants of at least 3.5 mm. in width and 10 mm. in length, leaving one millimeter of buccal and lingual plate and at least 2 mm. of distance to the lower alveolar dental nerve.

● Patients willing to attend all checkup visits at the School of Dentistry.

● Patients willing to provide informed consent to take part in the study.

- Exclusion criteria:

● Unmanaged psychiatric diseases.

● Unmanaged systemic disease.

● Presence of active infection or inflammation.

● Patients with previous history of radio and/or chemotherapy treatment.

● Patients with previous history of oral or intravenous bisphosphonates.

● Pregnancy.

● Smokers consuming more than 10 cigarettes per day.

● Clinical situations of post-extraction implants.

● Need to place implants with sinus lift procedures and/or in regenerated ridges/sockets.

- Pre-surgical protocol

Patient medical charts were filled out registering personal data, medical health and buccodental status. Extra-oral and intraoral pictures were taken. Study cast models were fabricated for each patient with alginate, and a diagnostic wax-up was made; later, a CT scan was made with a radiological guide. Implant planning was carried out using Physioplant™ software (PHYSIOPLANT SRL, Roncadelle, Italy).

Before surgery patients were administered with:

● Oral prophylaxis two weeks before surgery, in cases of a plaque index >20%

● Clorhexidine (0.12%) rinses twice daily for 45 seconds, from three days before surgery.

● Antibiotic therapy, amoxicillin 2g taken orally, one hour before treatment, clindamycin 600 mg administered one hour before surgery.

- Surgical procedure

All patients were treated under local anesthesia (Articaine 40 mg/0.01mg epinephrine). Incisions were performed as required by each particular situation. A full thickness flap was raised if needed and implant surgery was performed using a surgical guide, with total or partial guidance, prepared from the diagnostic wax-up. Two different implants macro-design were used in the study: A. Cylindrical shaped Implants (CI) Astra® Osseospeed (AstraTech Implant System, Dentsply Implants, Mölndal, Sweden) and Conical shaped Implants (TI) Mis® Seven (MIS®, Medical implants System, Israel). All implants were placed according to the manufacturers’ standard protocol. After implant placement, implant primary stability was measured by means of insertion torque (ITv) and ISQ value (ISQv) from Osstell™ Mentor (ISQv) (Integration Diagnostic Ltd., Goteborg, Sweden).

- Insertion torque:

At the moment of implant placement, an initial insertion torque of 20 Ncm was established, increasing by 5 Ncm if needed until total implant insertion had been achieved; in case of any exposed threads, a hand-wrench was used to achieve total insertion of the implant, in which case torque values were recorded as 50 N/cm.

- Resonance frequency analysis (RFA).

Resonance frequency measurements were recorded using Osstell™ Mentor (ISQv) (Integration Diagnostic Ltd., Goteborg, Sweden). A Smartpeg™ (Integration Diagnostic Ltd., Goteborg, Sweden) was attached to each implant with 5 Ncm. Smartpegs™ were used only once and were chosen in relation to the implant brand and connection. Each implant was measured twice, from two different angles, around 90 degrees apart, and parallel to the crestal line. The highest value was registered, and other values rejected. The Smartpeg™ was then removed and the area sutured with mono filament suture 4/0. To prevent early postoperative complications, antibiotics were prescribed: 500mg amoxicillin every 8 hours for 6 days post-surgery; in the case of a penicillin allergy, 300 mg clindamycin was administered every 6 hours for 5 days after surgery. Non-steroidal anti-inflammatory drugs (NSAIDs) were administered every 8 hours as needed. Postoperative instructions were given to the patient. Sutures were removed one week after surgery.

- Statistical Analysis:

Statistical analysis was performed using SPSS® 21.0 software (SPSS, Chicago, IL, USA). Values were expressed as the means ± standard deviation. Implant design (conical or cylindrical) was compared with the primary stability values of resonance frequency (ISQ) and insertion torque (Ncm), in both the mandible and maxilla. Normality was checked using the Shapiro-Wilk test. As the distribution of data was not normal, the Mann-Whitney test was applied. This test uses median values rather than means to perform comparative analysis of quantitative and qualitative variables. The Kruskal-Wallis test was used to analyze correlation between implant diameter and primary stability. The significance level was set at *p*=0.05.

## Results

**Figure 1:**
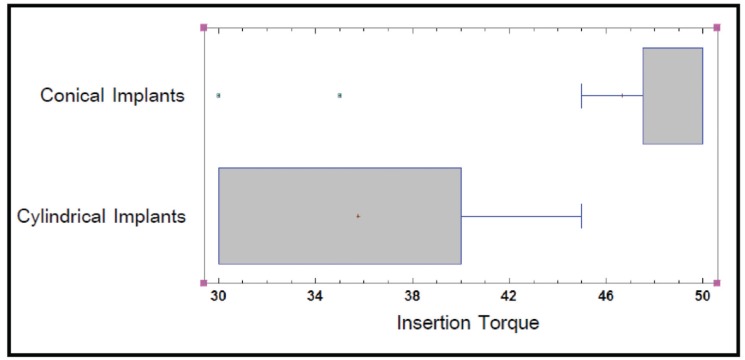
Insertion Torque Value by implant in the Mandible.

Data for the 47 implants tested in the study are expressed in  Table 1. In the mandible, the mean ISQ value for tapered implants was 71.67±5.16 and for cylindrical implants 57.15±4.83. (*p*=0.01). Insertion torque was 46.67±6.85 Ncm for tapered implants and 35.77±6.72 Ncm for cylindrical implants (*p*=0.01) (Fig. 1). 

**Table 1:**
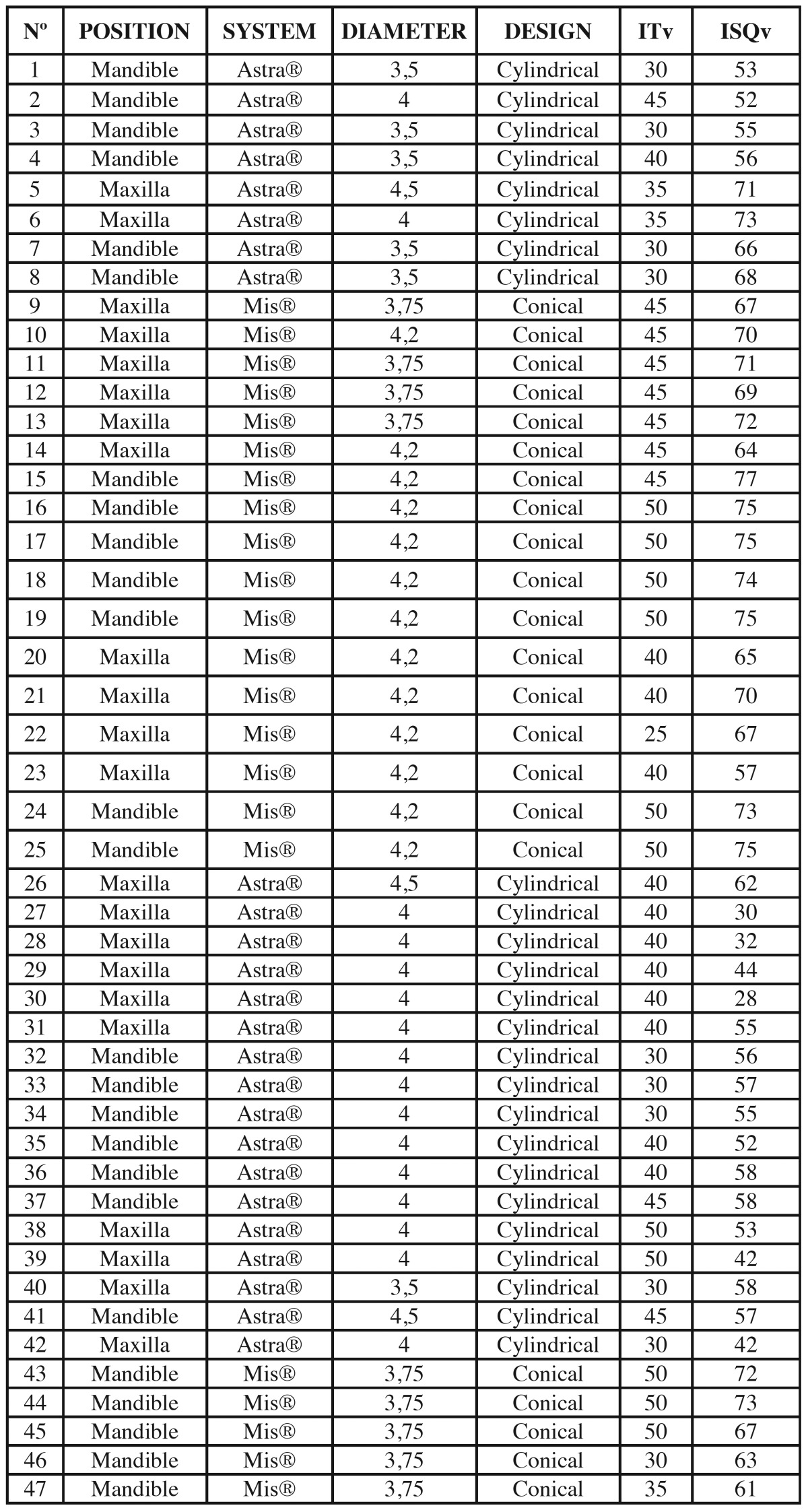
Data Collected.

**Figure 2:**
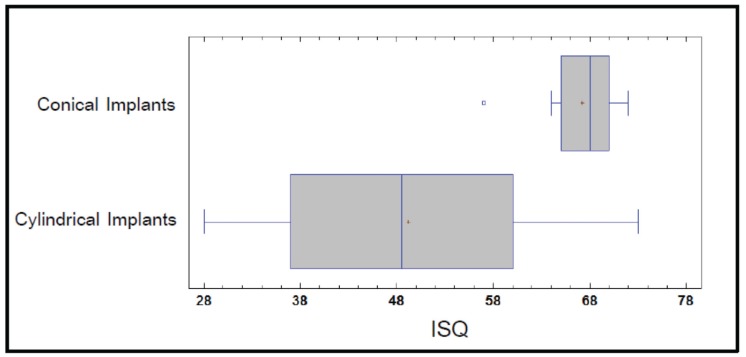
ISQ by implant in the Maxilla.

In the maxilla, the mean ISQ value for tapered implants was 67.2±4.42 and for cylindrical implants it was 49.17±15.30 (*p*=0.01) (Fig. 2). Mean insertion torque for tapered implants in the maxilla was 41.5±6.26 Ncm and 39.17±6.34 Ncm for cylindrical implants (*p*>0.05).

**Figure 3:**
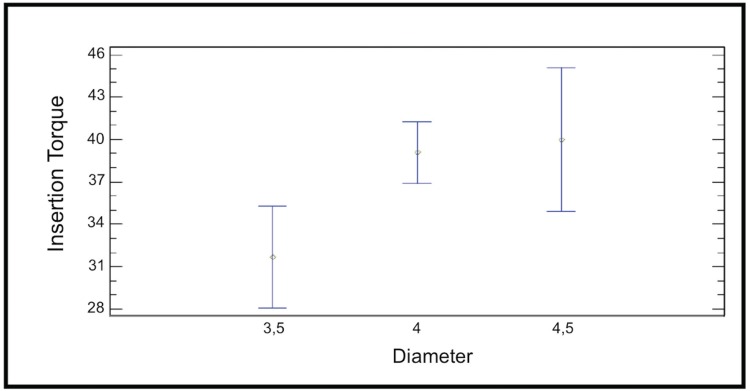
Relation between Insertion Torque value (ITv) and implant diameter in cylindrical implants group.

The Mann-Whitney test was used to determinate the influence of diameter on the ISQ and insertion torque of conical implants. For 3.75 mm diameter implants mean ISQ was 68.33±4.21 and mean insertion torque was 43.89±6.97 (*p*>0.05). For 4.2 mm diameter implants mean ISQ was 70.54±5.84 and mean insertion torque was 44.62±7.20 (*p*>0.05). The results showed no significant statistical relation between implant diameter and primary stability in conical design implants. The Kruskall-Wallis test was applied to the group of cylindrical implants. In this case, the *p*-value for the ISQ variable was 0.03 and for the insertion torque *p*=0.04. So for cylindrical design implants there is a significant statistical relation between diameter and primary stability (Fig. 3).

## Discussion

In the present study the ISQ and insertion torque values were higher in the mandible than in the maxilla, except for the insertion torque of cylindrical implants. The results suggest that conical implants achieve higher ISQ values (ISQv) (*p*=0.01) and insertion torque values (ITv) (*p*=0.01) than cylindrical design implants in the mandible. In the maxilla, tapered implants had higher ISQ values (ISQv) (*p*=0.01), although conical implants had higher insertion torque than cylindrical design implants, However no statistically significant differences were found (*p*>0.05).

In agreement with these results, Menicucci *et al*. (11) recently compared conical and cylindrical implant designs, finding higher insertion torque values for tapered implants (31.5 Ncm), compared with straight-walled implants (25.5 Ncm) (*p*=0.05). In another study, conducted by O’Sullivan *et al*. (12) in an animal model, tapered implants showed higher ISQ values and insertion torque compared with standard Bränemark implants. Similar results were obtained by this author and co-workers (13) in another human cadaver study. An animal study comparing different implant designs, concluded that tapered implants showed significantly higher stability than other conventional cylindrical implants (14). Rokn *et al*. (15) suggested that tapered implants exert more lateral compressive force on the bony walls surrounding the implant, so in areas with inadequate bone quality and quantity, the use of tapered implants is recommended to achieve better primary stability. In the present study, implants with exposed threads and with inserion torque over 50 Ncm were inserted with the wrench and were quantified as ITv of 50 Ncm, this fact might have distorted the results.

Regarding RFA analysis, recent studies performed with implants placed in artificial bone blocks or in animal models, have concluded that conical implants show significantly higher ISQ values compared to cylindrical implants (16-18). The results of a clinical trial performed by Dos Santos *et al*. (5) found higher insertion torque for conical implants than cylindrical implants; similar results were reported by Friberg *et al*. (19) Sakoh *et al*. (20) compared primary stability *in vitro* of two implants of different macro-design, one conical and the other cylindrical; in agreement with the present results, the conical implants showed significantly higher insertion torque, but did not show higher ISQ values. This also agrees with an animal study by Al-Nawas et al. (21). Contrary to the results of the present study, Bilhan *et al*. (22) assessed conical and cylindrical implants in an animal model; the authors found significantly higher insertion torque and ISQ values with the cylindrical implants. These differences might be explained by the fact that the implants were placed in cancellous bone and furthermore, the cylindrical implants were partially tapered.

The Osstell™ Mentor (Integration Diagnostic Ltd., Goteborg, Sweden) provides information about the stiffness of the implant-bone junction, while insertion torque is a mechanical parameter that measures cutting resistance. This difference may explain why in the maxilla no correlation was found between these two primary stability assessment parameters. Moreover, in addition to macro-design, other differences between implants may influence the results. For some researchers, in addition to macro-design (conical/cylindrical), surface treatment is also an important factor (23,24).

A secondary objective of this study was to determine the relationship between implant diameter and primary stability. For conical implants, no relation was found between diameter (3.75 mm or 4.2 mm) and ISQ values and insertion torque (*p*>0.05). However, there was a tendency for primary stability values to improve with the larger implant diameter. This finding is in agreement with an animal study by Ohta *et al*. (8) who showed that although the diameter of Replace Selected® tapered implants did not have a significant effect on ISQ values measured with the Osstell™ Mentor (Integration Diagnostic Ltd., Goteborg, Sweden), there was a tendency towards increased ISQ when implant diameter increased. Contrary to our results, an *in vitro* study of tapered implants by Tözum *et al*. (25) found a mean ISQ values of 78.7±0.6 and 81.6±0.6 for 3.75 mm diameter and for 4.2 mm diameter, respectively (*p*<0.05). More recent clinical and *in vitro* studies have shown that narrow platform implants present significantly lower ISQ values in comparison with regular and wide platform implants; however, no significant differences have been observed when comparing ISQ values (16).

In the group of cylindrical design implants, a statistically significant relationship between diameter (3.5 mm, 4 mm and 4.5 mm), insertion torque and ISQ values was found. However, it must be emphasized that the sample was not homogeneous, because 6 implants had a diameter of 3.5 mm, 16 had a diameter of 4 mm and 3 had a diameter of 4.5 mm, which may have distorted the results. Nevertheless, these results are in agreement with a clinical study by Park *et al*. (26) but do not agree with Han *et al*. (27) who concluded that implant diameter is not an important factor for ISQ values obtained by cylindrical implants. Barikani *et al*. (16) did not find significant differences in ISQ values between wide and regular diameter implants, but narrow implants showed significantly lower ISQ values than regular and wide implants.

Primary stability remains crucial to the success of immediate loading protocols. Recent research recommends an insertion torque value of between 32 and 50 Ncm, when performing immediate loading procedures (28-30). In the present study, only conical design implants group achieved insertion torque values over 32 Ncm across the entire sample. For this reason, conical implants should be the first option for ensuring adequate primary stability in immediate or early loading protocols.

## Conclusions

Within the limits of the present study, conical design implants achieved higher primary stability (measured by means of ISQ) and insertion torque values. For cylindrical implants, there was positive correlation between implant diameter and primary stability.

## References

[1] Leonhardt A, Gröndahl K, Bergström C, Lekholm U (2002). Long-term follow-up of osseointegrated titanium implants using clinical, radiographic and microbiological parameters. Clin Oral Implants Res.

[2] Meredith N (1998). Assessment of implant stability as a prognostic determinant. Int J Prosthodont.

[3] Javed F, Ahmed HB, Crespi R, Romanos GE (2013). Role of primary stability for successful osseointegration of dental implants: factors of influence and evaluation. Interv Med Appl Sci.

[4] Cehreli MC, Karasoy D, Akca K, Eckert SE (2009). Meta-analysis of methods used to assess implant stability. Int J Oral Maxillofac Implants.

[5] Dos Santos MV, Elias CN, Cavalcanti-Lima JH (2011). The effects of superficial roughness and design on the primary stability of dental implants. Clin Implant Dent Relat Res.

[6] Meredith N, Alleyne D, Cawley P (1996). Quantitative determination of the stability of the implant-tissue interface using resonance frequency analysis. Clin Oral Implants Res.

[7] Sennerby L, Meredith N (2008). Implant stability measurements using resonance frequency analysis: biological and biomechanical aspects and clinical implications. Periodontol 2000.

[8] Ohta K, Takechi M, Minami M, Shigeishi H, Hiraoka M, Nishimura M (2010). Influence of factors related to implant stability detected by wireless resonance frequency analysis device. J Oral Rehabil.

[9] Monje A, Suarez F, Garaicoa CA, Monje F, Galindo-Moreno P, García-Nogales A (2014). Effect of location on primary stability and healing of dental implants. Implant Dent.

[10] Romanos GE, Ciornei G, Jucan A, Malmstrom H, Gupta B (2014). In vitro assessment of primary stability of straumann implant designs. Clin Implant Dent Relat Res.

[11] Menicucci G, Pachie E, Lorenzetti M, Migliaretti G, Carossa S (2012). Comparison of primary stability of straight-walled and tapered implants using an insertion torque device. Int J Prosthodont.

[12] O'Sullivan D, Sennerby L, Meredith N (2004). Influence of implant taper on the primary and secondary stability of osseointegrated titanium implants. Clin Oral Implants Res.

[13] O'Sullivan D, Sennerby L, Meredith N (2000). Measurements comparing the initial stability of five designs of dental implants: a human cadaver study. Clin Implant Dent Relat Res.

[14] Toyoshima T, Tanaka H, Ayukawa Y, Howashi M, Masuzaki T, Kiyosue T (2015). Primary Stability of a Hybrid Implant Compared with Tapered and Cylindrical Implants in an Ex Vivo Model. Clin Implant Dent Relat Res.

[15] Rokn A, Ghahroudi AR, Mesgarzadeh A, Miremadi A, Yaghoobi S (2011). Evaluation of stability changes in tapered and parallel wall implants: a human clinical trial. J Dent (Tehran).

[16] Barikani H, Rashtak S, Akbari S, Fard MK, Rokn A (2014). The effect of shape, length and diameter of implants on primary stability based on resonance frequency analysis. Dent Res J (Isfahan).

[17] Romanos GE, Basha-Hijazi A, Gupta B, Ren YF, Malmstrom H (2014). Role of clinician's experience and implant design on implant stability. An ex vivo study in artificial soft bones. Clin Implant Dent Rela Res.

[18] García-Vives N, Andrés-García R, Rios-Santos V, Fernández-Palacín A, Bullón-Fernández P, Herrero-Climent M (2009). In vitro evaluation of the type of implant bed preparation with osteotomes in bone type IV and its influence on the stability of two implant systems. Med Oral Patol Oral Cir Bucal.

[19] Friberg B, Jisander S, Widmark G, Lundgren A, Ivanoff CJ, Sennerby L (2003). One-year prospective three-center study comparing the outcome of a "soft bone implant" (prototype Mk IV) and the standard Brånemark implant. Clin Implant Dent Relat Res.

[20] Sakoh J, Wahlmann U, Stender E, Nat R, Al-Nawas B, Wagner W (2006). Primary stability of a conical implant and a hybrid, cylindric screw-type implant in vitro. Int J Oral Maxillofac Implants.

[21] Al-Nawas B, Wagner W, Grötz KA (2006). Insertion torque and resonance frequency analysis of dental implant systems in an animal model with loaded implants. Int J Oral Maxillofac Implants.

[22] Bilhan H, Geckili O, Mumcu E, Bozdag E, Sünbüloğlu E, Kutay O (2010). Influence of surgical technique, implant shape and diameter on the primary stability in cancellous bone. J Oral Rehabil.

[23] Javed F, Romanos GE (2015). Role of implant diameter on long-term survival of dental implants placed in posterior maxilla: a systematic review. Clin Oral Investig.

[24] Dagher M, Mokbel N, Jabbour G, Naaman N (2014). Resonance frequency analysis, insertion torque, and bone to implant contact of 4 implant surfaces: comparison and correlation study in sheep. Implant Dent.

[25] Tözüm TF, Turkyilmaz I, Bal BT (2010). Initial stability of two dental implant systems: influence of buccolingual width and probe orientation on resonance frequency measurements. Clin Implant Dent Relat Res.

[26] Park JC, Ha SR, Kim SM, Kim MJ, Lee JB, Lee JH (2010). A randomized clinical 1-year trial comparing two types of non-submerged dental implants. Clin Oral Implants Res.

[27] Han J, Lulic M, Lang NP (2010). Factors influencing resonance frequency analysis assessed by Osstell mentor during implant tissue integration: II. Implant surface modifications and implant diameter. Clin Oral Implants Res.

[28] Wang HL, Ormianer Z, Palti A, Perel ML, Trisi P, Sammartino G (2006). Consensus conference on immediate loading: the single tooth and partial edentulous areas. Implant Dent.

[29] Aparicio C, Rangert B, Sennerby L (2003). Immediate/early loading of dental implants: a report from the Sociedad Espa-ola de Implantes World Congress consensus meeting in Barcelona, Spain, 2002. Clin Implant Dent Relat Res.

[30] Misch CE, Wang HL, Misch CM, Sharawy M, Lemons J, Judy KW (2004). Rationale for the application of immediate load in implant dentistry: part II. Implant Dent.

